# Disability and Recurrent Stroke Among Participants in Stroke Prevention Trials

**DOI:** 10.1001/jamanetworkopen.2024.23677

**Published:** 2024-07-19

**Authors:** Adam de Havenon, Catherine Viscoli, Dawn Kleindorfer, Heidi Sucharew, Alen Delic, Christopher Becker, David Robinson, Shadi Yaghi, Vivian Li, Maarten G. Lansberg, Steven C. Cramer, Eva A. Mistry, Daniel F. Sarpong, Scott E. Kasner, Walter Kernan, Kevin N. Sheth

**Affiliations:** 1 Department of Neurology, Center for Brain and Mind Health, Yale University School of Medicine, New Haven, Connecticut; 2 Department of Internal Medicine, Yale University School of Medicine, New Haven, Connecticut; 3 Department of Neurology, University of Michigan, Ann Arbor; 4 Department of Emergency Medicine, University of Cincinnati, Cincinnati, Ohio; 5 Department of Neurology, University of Utah, Salt Lake City; 6 Department of Neurology, Brown University, Providence, Rhode Island; 7 Department of Neurology, Stanford University, Palo Alto, California; 8 Department of Neurology, University of California and California Rehabilitation Institute, Los Angeles; 9 Department of Neurology and Rehabilitation Medicine, University of Cincinnati, Cincinnati, Ohio; 10 Department of Neurology, University of Pennsylvania, Philadelphia

## Abstract

**Importance:**

Stroke secondary prevention trials have disproportionately enrolled participants with mild or no disability. The impact of this bias remains unclear.

**Objective:**

To investigate the association between poststroke disability and the rate of recurrent stroke during long-term follow up.

**Design, Setting, and Participants:**

This cohort study is a post hoc analysis of the Prevention Regimen For Effectively Avoiding Second Strokes (PRoFESS) and Insulin Resistance Intervention After Stroke (IRIS) secondary prevention clinical trial datasets. PRoFESS enrolled patients from 2003 to 2008, and IRIS enrolled patients from 2005 to 2015. Data were analyzed from September 23, 2023, to May 16, 2024.

**Exposure:**

The exposure was poststroke functional status at study baseline, defined as modified Rankin Scale (mRS; range, 0-5; higher score indicates more disability) score of 0 vs 1 to 2 vs 3 or greater.

**Main Outcomes and Measures:**

The primary outcome was recurrent stroke. The secondary outcome was major cardiovascular events (MACE), defined as recurrent stroke, myocardial infarction, new or worsening heart failure, or vascular death.

**Results:**

A total of 20 183 PRoFESS participants (mean [SD] age, 66.1 [8.5] years; 12 931 [64.1%] male) and 3265 IRIS participants (mean [SD] age, 62.7 [10.6] years; 2151 [65.9%] male) were included. The median (IQR) follow-up was 2.4 (1.9-3.0) years in PRoFESS and 4.7 (3.2-5.0) years in IRIS. In PRoFESS, the recurrent stroke rate was 7.2%, among patients with an mRS of 0, 8.7% among patients with an mRS of 1 or 2, and 10.6% among patients with an mRS of 3 or greater (χ^2^_2_ = 27.1; *P* < .001); in IRIS the recurrent stroke rate was 6.4% among patients with an mRS of 0, 9.0% among patients with an mRS of 1 or 2, and 11.7% among patients with an mRS of 3 or greater (χ^2^_2_ = 11.1; *P* < .001). The MACE rate was 10.1% among patients with an mRS of 0, 12.2% among patients with an mRS of 1 or 2, and 17.2% among patients with an mRS of 3 or greater (χ^2^_2_ = 103.4; *P* < .001) in PRoFESS and 10.9% among patients with an mRS of 0, 13.3% among patients with an mRS of 1 or 2, and 15.3% among patients with an mRS of 3 or greater (χ^2^_2_ = 5.8; *P* = .06) in IRIS. Compared with patients with an mRS of 0, patients with an mRS of 3 or greater had increased hazard for recurrent stroke in PRoFESS (hazard ratio [HR], 1.63; 95% CI, 1.38-1.92; *P* < .001) and in IRIS (HR, 1.91; 95% CI, 1.28-2.86; *P* = .002). There was also increased hazard for MACE in PRoFESS (HR, 1.90; 95% CI, 1.66-2.18; *P* < .001) and in IRIS (HR, 1.45; 95% CI, 1.03-2.03; *P* = .03).

**Conclusions and Relevance:**

This cohort study found that higher baseline poststroke disability was associated with increased rates of recurrent stroke and MACE. Including more patients with greater baseline disability in stroke prevention trials may improve the statistical power and generalizability of these studies.

## Key Points

**Question:**

Is the level of poststroke disability associated with the rate of recurrent stroke and major cardiovascular events during long-term follow-up?

**Findings:**

In this cohort study using data from 2 randomized clinical trials of secondary stroke prevention, higher baseline poststroke disability (measured by the modified Rankin Scale) was associated with increased rates of recurrent stroke and major cardiovascular events.

**Meaning:**

These findings suggest that including more patients with greater baseline disability in stroke prevention trials may improve the statistical power and generalizability, potentially leading to better-informed secondary prevention strategies.

## Teaser

This cohort study assesses the association of poststroke disability with risk of recurrent stroke and major cardiovascular events among participants in 2 randomized clinical trials of secondary stroke prevention.

## Introduction

Secondary prevention trials in ischemic stroke populations have disproportionately enrolled participants with mild or no disability related to the qualifying stroke.^1^ Among recent large trials, both NAVIGATE ESUS^2^ and RE-SPECT ESUS^3^ enrolled patients with a median (IQR) modified Rankin Scale (mRS) and National Institutes of Health Stroke Scale (NIHSS) scores of 1 (0-2). Both trials excluded individuals with an mRS of 4 or greater at baseline. When trials predominantly enroll participants with less disability, their results may not be generalizable to most survivors of stroke who have long-term moderate or severe disability.^4,5,6,7^ Furthermore, the risk of recurrent stroke may be hypothetically lower in individuals with less poststroke disability, which would reduce the power to detect a treatment effect, and there may be differences in gender, race, or ethnicity by disability status. To test this hypothesis, we performed post hoc analyses of the Prevention Regimen For Effectively Avoiding Second Strokes (PRoFESS) and Insulin Resistance Intervention After Stroke (IRIS) trials.^8,9^ We selected these trials because they permitted enrollment of individuals with an mRS score of 4.

## Methods

### Data Source and Acquisition of Cohort

This is a post hoc analysis of the PRoFESS and IRIS trials. PRoFESS data were obtained from Vivli, a consortium of anonymized clinical study data. Requests to access the dataset from qualified researchers may be sent to Vivli.^10^ IRIS data are publicly available from the National Institutes of Health National Institute of Neurological Disorders and Stroke.^11^ Local institutional review board approval and informed consent were not required for the analyses of either deidentified dataset per the Common Rule. We conformed to the Strengthening the Reporting of Observational Studies in Epidemiology (STROBE) reporting guideline for reporting of this cohort study.

From 2003 to 2008, PRoFESS enrolled and followed 20 332 patients aged at least 55 years with an ischemic stroke within the past 90 days or patients aged 50 to 54 years and/or 90 to 120 days from stroke onset with at least 2 of the following: diabetes, hypertension, current smoker, obesity, previous vascular disease or end organ damage, or hyperlipidemia.^8,12^ Exclusion criteria included hemorrhagic stroke at entry, mRS score of 5 at baseline, recent coronary artery disease or major surgery, severe hepatic or kidney insufficiency, and uncontrolled hypertension or hypotension. PRoFESS had a 2 × 2 factorial design testing aspirin and dipyridamole vs clopidogrel and telmisartan vs placebo. The median time from the qualifying stroke to randomization was 15 days, and PRoFESS followed participants for a mean of 2.5 years.

From 2005 to 2015, IRIS enrolled and followed 3876 patients aged at least 40 years with an ischemic stroke or transient ischemic attack within the past 180 days and insulin resistance based on the Homeostasis Model Assessment. Individuals with diabetes, as defined by the American Diabetes Association’s recommendations, were excluded. In addition, those with an mRS score of 5 at baseline, heart failure, active liver disease, or bladder cancer were also excluded. Participants were randomized to receive either placebo or pioglitazone (adjusted up to 45 mg daily) and were followed for a median of 4.7 years.

### Inclusion, Exposure, and Outcomes

We included individuals enrolled in either trial with ischemic stroke as the qualifying event, an available mRS at enrollment, nonmissing baseline demographic data, and documented follow-up past the baseline visit. The primary study exposure was poststroke functional status, defined as an mRS score of 0 (no symptoms) vs 1 to 2 (functional independence) vs 3 or greater (moderate to severe dependence).^13^ As sensitivity analyses, we defined the exposure as mRS score of 0 to 1 vs 2 vs 3 or greater and poststroke NIHSS score of 0 vs 1 to 2 vs 3 or greater. We used the NIHSS because it corresponds to impairment status as opposed to disability.^14^ The study exposures in PRoFESS are measured at the baseline and randomization study visit. In IRIS, they are measured at a screening visit, which was a median (IQR) of 58 (31-100) days after stroke onset.

The primary outcome was recurrent stroke of any type. The secondary outcome was major cardiovascular events (MACE), defined as recurrent stroke, myocardial infarction, new or worsening heart failure, or vascular death. In the PRoFESS subgroup without a primary or secondary event, we examined rates of any study drug discontinuation, nonadherence with the protocol leading to discontinuation, premature trial termination, and nonvascular death as tertiary outcomes. The intent of the tertiary outcomes was to measure factors that could affect trial feasibility. All outcome events eligible for adjudication happened after randomization. Study events were coded according to the trials’ stated definitions.^8,9^

### Statistical Analysis

The trial cohorts were analyzed separately. To determine whether poststroke functional status was associated with recurrent stroke or MACE, we constructed Cox regression models. Censoring occurred for primary or secondary outcome events, trial completion, loss to follow-up, or all-cause death. We report unadjusted hazard ratios (HRs) in the primary analysis. We also report HRs adjusted for baseline age, self-reported gender, self-reported race and ethnicity (categorized as Asian [non-Hispanic], Black [non-Hispanic], Hispanic, White [non-Hispanic], and other [any other self-reported racial or ethnic category or unknown]), geographic region or country of enrollment, current smoking, atrial fibrillation, hypertension, hyperlipidemia, prior stroke, randomization group, days from the qualifying stroke to enrollment, and Trial of ORG 10172 in Acute Stroke Treatment (TOAST) classification of the qualifying stroke.^15^ In PRoFESS, we also adjusted for diabetes status, but not in IRIS because it was an exclusion criterion.

We assessed the proportional hazards assumption of each model based on the Schoenfeld residuals. If the global test of the proportional hazards assumption was violated, we performed a separate test for each covariate to identify the violations at a level of *P* < .10. We then used a stratified Cox model to allow for nonproportionality of the covariate that had violations.^16^ Observations with equal values of the strata variables are placed within the same strata. Stratified estimates are then obtained using equal coefficients across strata but with a baseline hazard unique to each stratum. We did not impute data and assumed missing data were missing at random. *P* values were 2-sided, and statistical significance was set at *P* < .05. All analyses was conducted using Stata software version 18.0 (StataCorp). We performed the analysis from September 23, 2023, to May 16, 2024.

## Results

The derivation of the PRoFESS cohort is presented in eFigure 1 in Supplement 1 and of the IRIS cohort in eFigure 2 in Supplement 1. In the PRoFESS cohort, we included 20 183 individuals (mean [SD] age, 66.1 [8.5] years; 12 931 [64.1%] male), and in the IRIS cohort, we included 3265 individuals (mean [SD] age, 62.7 [10.6] years; 2151 [65.9%] male). The PRoFESS cohort included 6671 Asian (non-Hispanic) participants (33.0%), 803 Black (non-Hispanic) participants (4.0%), 981 Hispanic participants (4.9%), 11 561 White (non-Hispanic) participants (57.3%), and 167 participants who identified as other race or ethnicity (0.8%); IRIS included 363 Black (non-Hispanic) participants (11.1%), 128 Hispanic participants (3.9%), 2616 White (non-Hispanic) participants (80.1%), and 151 participants who identified as other race or ethnicity (4.9%). The median (IQR) duration of follow-up was 2.4 (1.9-3.0) years in PRoFESS and was 4.7 (3.2-5.0) years in IRIS. In PRoFESS, 2834 participants (14.0%) had an mRS score of 0, 12 570 participants (62.3%) had an mRS score of 1 or 2, and 4779 participants (23.7%) had an mRS score of 3 or more; in IRIS 1096 participants (33.6%) had an mRS score of 0, 1862 participants (57.0%) had an mRS score of 1 or 2, and 307 participants (9.4%) had an mRS score of 3 or greater. Baseline demographics are presented after stratification by mRS categories for PRoFESS in Table 1 and for IRIS in Table 2. In both trials, greater poststroke disability was associated with female gender; Black (non-Hispanic) race and ethnicity in IRIS and Hispanic ethnicity in PRoFESS; shorter time from qualifying stroke to enrollment; prior stroke; hypertension; and large artery atherosclerosis. In PRoFESS, 35.7% of participants with an mRS score of 0 were female vs 34.7% of participants with an mRS score of 1 or 2 vs 39.4% of participants with an mRS score of 3 or greater (χ^2^_2_ = 34.3; *P* < .001), and in IRIS, 32.3% of participants with an mRS score of 0 were female vs 34.2% of participants with an mRS score of 1 or 2 vs 40.1% of participants with an mRS score of 3 or greater (χ^2^_2_ = 6.5; *P* = .04). Both cohorts had significant differences in race and ethnicity by mRS score (Table 1 and Table 2).

**Table 1.** Baseline Demographics for the Prevention Regimen for Effectively Avoiding Second Strokes Cohort Stratified by mRS

Characteristic	Participants, No. (%)				*P* value^a^
	Total (n = 20 183)	mRS score			
		0 (n = 2834)	1-2 (n = 12 570)	3-5 (n = 4779)	
Age, mean (SD), y	66.1 (8.5)	66.8 (8.4)	65.7 (8.4)	66.7 (8.7)	<.001
Gender					
Female	7252 (35.9)	1012 (35.7)	4356 (34.7)	1884 (39.4)	<.001
Male	12 931 (64.1)	1822 (64.3)	8214 (65.4)	2895 (60.6)	
Race and ethnicity					
Asian (non-Hispanic)	6671 (33.0)	439 (15.5)	4024 (32.0)	2208 (46.2)	<.001
Black (non-Hispanic)	803 (4.0)	122 (4.3)	492 (3.9)	189 (4.0)	
Hispanic	981 (4.9)	101 (3.6)	589 (4.7)	291 (6.1)	
White (non-Hispanic)	11 561 (57.3)	2145 (75.7)	7349 (58.4)	2067 (43.3)	
Other or unknown	167 (0.8)	27 (1.0)	116 (0.9)	24 (0.5)	
Prior stroke	3670 (18.2)	348 (12.3)	2212 (17.6)	1110 (23.2)	<.001
Current smoker	4283 (21.2)	523 (18.5)	2772 (22.1)	988 (20.7)	<.001
Hypertension	14 932 (74.0)	1958 (69.1)	9338 (74.3)	3636 (76.1)	<.001
Hyperlipidemia	9448 (46.8)	1553 (54.8)	5973 (47.5)	1922 (40.2)	<.001
Atrial fibrillation	529 (2.6)	50 (1.8)	322 (2.6)	157 (3.3)	<.001
Diabetes	5705 (28.3)	584 (20.6)	3468 (27.6)	1653 (34.6)	<.001
Trial group					
AP	5053 (25.0)	695 (24.5)	3146 (25.0)	1212 (25.4)	.85
AT	5026 (24.9)	715 (25.2)	3103 (24.7)	1208 (25,3)	
CP	5059 (25.1)	712 (25.1)	3146 (25.0)	1201 (25.1)	
CT	5045 (25.0)	712 (25.1)	3175 (25.3)	1158 (24.2)	
Days from stroke to enrollment	26.9 (27.4)	35.5 (30.3)	26.9 (27.2)	22.0 (24.5)	<.001
Region					
North America	4907 (24.3)	1096 (38.7)	2973 (23.7)	838 (17.6)	<.001
Latin America	1049 (5.2)	129 (4.5)	597 (4.8)	323 (6.8)	
Asia	6938 (34.4)	457 (16.1)	4246 (33.7)	2246 (47.0)	
Europe	6942 (34.4)	1062 (37.4)	4578 (36.3)	1313 (27.5)	
Africa	78 (0.4)	9 (0.3)	46 (0.4)	23 (0.5)	
Oceania	269 (1.3)	83 (2.9)	149 (1.2)	37 (0.8)	
Stroke subtype					
Large artery	5778 (28.6)	620 (21.9)	3216 (25.5)	1942 (40.6)	<.001
Cardioembolic	361 (1.8)	56 (2.0)	197 (1.6)	108 (2.3)	
Small vessel	10 508 (52.1)	1523 (53.7)	6929 (55.2)	2056 (43.0)	
Other	414 (2.1)	86 (3.0)	256 (2.0)	72 (1.5)	
Cryptogenic	3122 (15.5)	549 (19.4)	1972 (15.7)	601 (12.6)	

Abbreviations: AP, aspirin and dipyridamole plus placebo; AT, aspirin and dipyridamole plus telmisartan; CP, clopidogrel plus placebo; CT, clopidogrel plus telmisartan; mRS, modified Rankin Scale.

^a^ Intergroup differences were tested with analysis of variance for continuous variables and the χ^2^ test for binary variables.

**Table 2.** Baseline Demographics for the Insulin Resistance Intervention After Stroke Cohort Stratified by Poststroke Categories of mRS

Characteristic	Participants, No. (%)				*P* value^a^
	Total (n = 3265)	mRS score			
		0 (n = 1096)	1-2 (n = 1862)	3-5 (n = 307)	
Age, mean (SD), y	62.7 (10.6)	62.9 (10.4)	62.7 (10.6)	62.2 (11.3)	.62
Gender					
Female	1114 (34.1)	354 (32.3)	637 (34.2)	123 (40.1)	.04
Male	2151 (65.9)	742 (67.7)	1225 (65.8)	184 (59.9)	
Race and ethnicity					
Black (non-Hispanic)	363 (11.1)	83 (7.6)	230 (12.4)	50 (16.3)	<.001
Hispanic	128 (3.9)	42 (3.8)	69 (3.7)	17 (5.5)	
White (non-Hispanic)	2616 (80.1)	915 (83.5)	1474 (79.2)	227 (73.9)	
Other or unknown	151 (4.9)	56 (5.1)	89 (4.7)	13 (4.3)	
Prior stroke	405 (12.4)	97 (8.9)	252 (13.5)	56 (18.2)	<.001
Current Smoker	521 (16.0)	173 (15.8)	313 (16.8)	35 (11.4)	.06
Hypertension	2342 (71.7)	741 (67.6)	1375 (73.8)	226 (73.6)	<.001
Hyperlipidemia	2224 (68.1)	760 (69.3)	1261 (67.7)	203 (66.1)	.48
Atrial fibrillation	224 (6.9)	79 (7.2)	119 (6.4)	26 (8.5)	.35
Intervention group in trial	1638 (50.2)	548 (50.0)	930 (49.9)	160 (52.1)	.77
Days from stroke to enrollment	90.3 (45.5)	93.9 (45.4)	88.0 (44.9)	91.8 (48.3)	.003
Country					
United States	2227 (68.2)	712 (65.0)	1263 (67.8)	252 (82.1)	<.001
Canada	439 (13.4)	148 (13.5)	266 (14.3)	25 (8.1)	
Germany	126 (3.9)	48 (4.4)	77 (4.1)	1 (0.3)	
Israel	153 (4.7)	68 (6.2)	77 (4.1)	8 (2.6)	
Italy	43 (1.3)	23 (2.1)	16 (0.9)	4 (1.3)	
England	192 (5.9)	72 (6.6)	109 (5.9)	11 (3.6)	
Australia	85 (2.6)	25 (2.3)	54 (2.9)	6 (2.0)	
Stroke subtype					
Large artery	867 (26.6)	277 (25.3)	480 (25.8)	110 (35.8)	.001
Cardioembolic	256 (7.8)	91 (8.3)	133 (7.1)	32 (10.4)	
Small vessel	1026 (31.4)	328 (29.9)	615 (33.0)	83 (27.0)	
Other	90 (2.8)	38 (3.5)	49 (2.6)	3 (1.0)	
Cryptogenic	1008 (30.9)	356 (32.5)	575 (30.9)	77 (25.1)	
Multiple	18 (0.6)	6 (0.5)	10 (0.5)	2 (0.7)	

Abbreviation: mRS, modified Rankin Scale.

^a^ Intergroup differences tested with analysis of variance for continuous variables and the χ^2^ test for binary variables.

The recurrent stroke rate across mRS scores was 7.2% among participants with a score of 0, 8.7% among participants with a score of 1 or 2, and 10.6% among participants with a score of 3 or greater (χ^2^_2_ = 27.1; *P* < .001) in PRoFESS and 6.4% among participants with a score of 0, 9.0% among participants with a score of 1 or 2, and 11.7% among participants with a score of 3 or greater (χ^2^_2_ = 11.1; *P* < .001). In PRoFESS, the MACE rate was 10.1% among participants with a score of 0, 12.2% among participants with a score of 1 or 2, and 17.2% among participants with a score of 3 or greater (χ^2^_2_ = 103.4; *P* < .001), and in IRIS, the MACE rate was 10.9% among participants with a score of 0, 13.3% among participants with a score of 1 or 2, and 15.3% among participants with a score of 3 or greater (χ^2^_2_ = 5.8; *P* = .06). In both trials, we saw consistent increases in the hazard for recurrent stroke and MACE in unadjusted analyses (Table 3; eTable 1 in Supplement 1). In PRoFESS, compared with an mRS score of 0, an mRS score of 3 or greater was associated with 63% increased hazard of recurrent stroke (HR, 1.63; 95% CI, 1.38-1.92; *P* < .001), while in IRIS, the hazard was increased by 91% (HR, 1.91; 95% CI, 1.28-2.86; *P* = .002). The hazard for MACE in participants with an mRS score of 3 or greater, compared with participants with an mRS score of 0, was significantly increased in PRoFESS (HR, 1.90; 95% CI, 1.66-2.18; *P* < .001) and in IRIS (HR, 1.45; 95% CI, 1.03-2.03; *P* = .03). Adjusted models retained statistical significance except for the MACE model in IRIS (Table 3; eTable 1 in Supplement 1). Kaplan-Meier curves for both outcomes in the PRoFESS cohort are presented in the Figure.

**Table 3.** Recurrent Stroke Outcomes in PRoFESS and IRIS by Poststroke Categories of mRS

mRS score	Stroke event rate, %	Unadjusted HR (95% CI)	*P* value	Adjusted HR (95% CI)^a^	*P* value
**PRoFESS**					
0 (n = 2834)	7.2	1 [Reference]	NA	1 [Reference]	NA
1-2 (n = 12 570)	8.7	1.27 (1.09-1.47)	.002	1.18 (1.01-1.37)	.03
≥3 (n = 4779)	10.6	1.63 (1.38-1.92)	<.001	1.36 (1.14-1.61)	<.001
**IRIS**					
0 (n = 1096)	6.4	1 [Reference]	NA	1 [Reference]	NA
1-2 (n = 1862)	9.0	1.44 (1.09-1.90)	.011	1.36 (1.02-1.80)	.03
≥3 (n = 307)	11.7	1.91 (1.28-2.86)	.002	1.79 (1.19-2.70)	.006

Abbreviations: IRIS, Insulin Resistance Intervention After Stroke; mRS, modified Rankin Scale; NA, not applicable; PRoFESS, Prevention Regimen For Effectively Avoiding Second Strokes.

^a^ Adjusted for baseline age, gender, race and ethnicity, region or country of enrollment, current smoking, atrial fibrillation, hypertension, hyperlipidemia, prior stroke, randomization group, days from qualifying stroke to enrollment, and Trial of ORG 10172 in Acute Stroke Treatment classification of the qualifying stroke. PRoFESS is also adjusted for diabetes.

**Figure.**
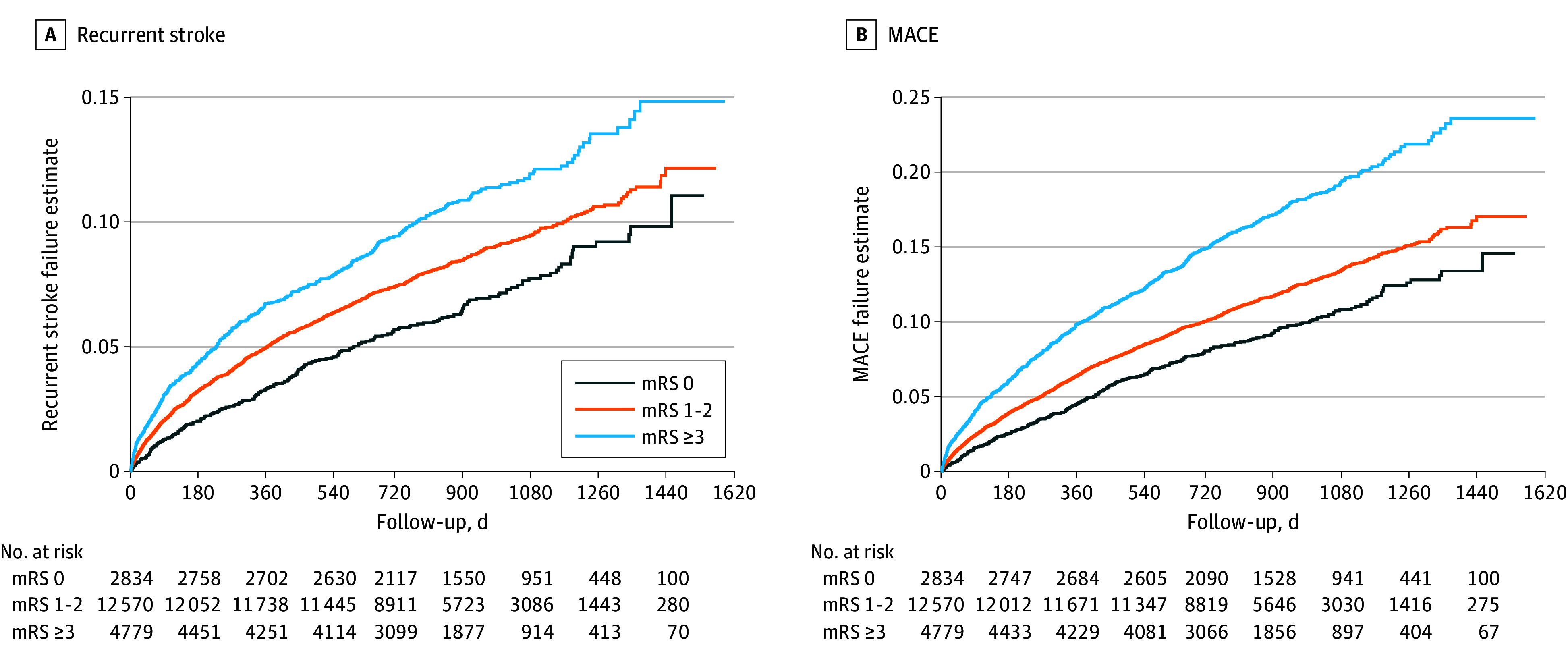
Kaplan-Meier Curves for Recurrent Stroke Events and Major Cardiovascular Events (MACE) in the Prevention Regimen for Effectively Avoiding Second Strokes Trial Cohort, Stratified by Poststroke Categories of the Modified Rankin Scale (mRS)

In the sensitivity analysis in which we used NIHSS instead of mRS, there were similar results, despite more than 40% of individuals being in discordant categories (rate of discordance: PRoFESS, 40.3%; IRIS, 44.4%) (eFigure 3 and eFigure 4 in Supplement 1). Nonetheless, using NIHSS, we again saw consistent increases in the hazard of recurrent stroke across the NIHSS categories (eTable 2 in Supplement 1). Adjusted models retained statistical significance, except for the MACE model in IRIS (eTable 3 in Supplement 1). These results were also consistent when the exposure was defined as mRS 0 to 1 vs 2 vs 3 or greater (eTable 4 and eTable 5 in Supplement 1).

The interaction terms between the treatment group and poststroke mRS were not significant for either PRoFESS or IRIS in the adjusted models fit to recurrent stroke nor in the models fit to MACE. In PRoFESS, among 17 576 individuals who did not have a primary or secondary event, the rate of any study drug discontinuation was 38.2% among participants with an mRS score of 0, 34.0% among participants with an mRS score of 1 or 2, and 37.2% among participants with an mRS score of 3 or greater (χ^2^_2_ = 26.3; *P* < .001). The rate of drug discontinuation due to nonadherence with the protocol was 6.7% among participants with an mRS score of 0, 7.8% among participants with an mRS score of 1 or 2, and 9.5% among participants with an mRS score of 3 or greater (χ^2^_2_ = 18.2; *P* < .001). The rate of premature trial termination was 4.4% among participants with an mRS score of 0, 3.5% among participants with an mRS score of 1 or 2, and 5.7% among participants with an mRS score of 3 or greater (χ^2^_2_ = 36.5; *P* < .001). The rate of nonvascular death was 2.5% among participants with an mRS score of 0, 2.2% among participants with an mRS score of 1 or 2, and 4.4% among participants with an mRS score of 3 or greater (χ^2^_2_ = 63.3; *P* < .001). In 174 individuals with an mRS score of 3 or greater who had a nonvascular death, the median (IQR) follow-up prior to death was 474 (210-704) days.

## Discussion

In this cohort study, we found a significant association between greater poststroke disability, as determined by the mRS, and increased risk of recurrent stroke and MACE in 2 large clinical trials of secondary stroke prevention. We also found that in both trials, greater baseline disability was associated with self-reported female gender and Hispanic ethnicity in PRoFESS and Black (non-Hispanic) race and ethnicity in IRIS. In the PRoFESS trial, participants with an mRS score of 3 or greater had recurrent stroke rate of 10.6% during follow-up, compared with 7.2% in participants with an mRS score of 0. Similar results were seen in IRIS, with a rate of 11.7% among participants with an mRS score of 3 or greater, compared with 6.4% among participants with an mRS score of 0. These findings persisted after adjusting for known vascular risk factors for recurrent stroke.

The robustness of these results was further demonstrated by our sensitivity analyses using a different cut point for mRS and NIHSS, a measure of impairment rather than disability. Despite more than 40% discordance between mRS and NIHSS categories, the association between greater poststroke impairment, as assessed by NIHSS, and increased risk of recurrent stroke and MACE remained consistent in both trials. This discordance of function (mRS) and impairment (NIHSS) is an interesting phenomenon that warrants additional research to explore what elements of poststroke disability and impairment are best captured by mRS vs NIHSS.^14^

We did not find that poststroke disability had a significant interaction with the trials’ treatment groups, indicating that treatment effects were comparable across the levels of poststroke disability. It is also important to note that the poststroke disability and impairment measures we used were measured at a median of 15 days in PRoFESS and 58 days in IRIS, indicating that our findings are valid at different time points in the initial recovery period after stroke.^17,18,19,20^

We are not aware of prior analyses that have specifically covered the topic of this analysis. An analysis of the Virtual International Stroke Trials Archive database showed that 90-day recurrent stroke rates were higher in participants with an mRS score of 4 or 5 at enrollment but did not have long-term follow-up.^1^ Several scores exist to estimate the long-term risk of recurrent stroke, including the Essen Stroke Risk Score and Stroke Prognosis Instrument, neither of which considers poststroke disability.^21,22,23,24^ This omission may be attributed to limitations in the datasets used to develop these scores.

The intent of this analysis, however, was not to demonstrate novel factors associated with recurrent stroke or MACE risk. Rather, we performed this analysis to help address the ramifications of the ableism bias^25^ that results in secondary stroke prevention trials predominantly enrolling survivors of stroke with no or minimal disability.^26,27^ Furthermore, patients with poststroke disability are a substantial subset (>50%) of the stroke survivor population^4,5,6,7^ and thus also need to be studied with respect to secondary prevention.

Our analysis also highlights the potential benefits of enrolling a higher proportion of participants with poststroke disability for improved trial statistical power. In addition, we found that individuals enrolled in PRoFESS and IRIS with higher poststroke disability were more likely to be female and underrepresented race or ethnicity. This suggests that enrolling more participants with higher levels of poststroke disability may also help stroke trials address longstanding generalizability issues caused by low levels of participation among female and Black and Hispanic individuals in stroke and cardiovascular trials.^28,29^

Individuals with disability are poorly represented in most clinical trial populations.^30^ A persistent concern is that disability could lead to issues with medication adherence, adherence to a protocol, or higher rates of loss to follow-up.^31^ Anticipating this concern, we performed an analysis in PRoFESS that found that participants with an mRS score of 3 or greater were less likely than those with an mRS score of 0 to discontinue the study drug for any reason and had trivial increases in the rate of protocol nonadherence, premature trial termination, or nonvascular death. Even the nonvascular death in participants with an mRS score of 3 or greater happened at a median of 474 days, thus providing ample data prior to censoring. Another concern is that individuals with poststroke functional impairment are often discharged to acute care or rehabilitation facilities, which could complicate trial procedures if not preemptively addressed during the protocol design phase.^32,33,34,35^

However, there still remains an important gap between reality for individuals with disabilities and researcher expectations, which can manifest in trial protocols that are difficult or impossible for individuals with disabilities to follow (eg, complicated consents or other study documentation, frequent in-person follow-up, long appointments with multiple locations for testing).^31,36^ Understanding this, it becomes imperative to incorporate codesign and community consultation when designing future secondary stroke trial protocols, ensuring that the experiences and needs of patients across all disability levels, genders, and races and ethnicities are represented.^37,38^ More importantly, adopting an inclusive approach in stroke trials aligns ethically with the social model of disability. This model challenges prevailing knowledge hierarchies that have historically marginalized specific groups, such as individuals with poststroke or neurologic disability.^39^

### Limitations

This study has some limitations. The participants from the PRoFESS and IRIS trials were mostly enrolled more than a decade ago (2003-2008 for PRoFESS and 2005-2015 for IRIS), which may introduce a bias from the changes in stroke prevention. However, a 2018 meta-analysis showed that the annual risk of recurrent stroke in prevention trials has remained stable at approximately 4.6% since 1980.^40^ Our study only included PRoFESS and IRIS participants with nonmissing data, which might introduce a selection bias. Furthermore, the mRS is a widely used measure of disability, but like any ordinal scale, it has limitations, including modest interobserver reliability and a relatively crude categorical nature.^41,42^

## Conclusions

The findings from this post hoc cohort study of the PRoFESS and IRIS clinical trial datasets underscore an important issue in ischemic stroke secondary prevention trials: the underrepresentation of participants with moderate to severe disability. Our results reveal a clear association between higher poststroke disability and an increased rate of recurrent strokes and MACE. This emphasizes the pressing need for a more inclusive enrollment strategy that encompasses the full spectrum of disability, ensuring both enhanced statistical power and broader generalizability of outcomes. Furthermore, by embracing a comprehensive enrollment approach, clinical trials would better mirror practical scenarios, offering more applicable insights to broader, more diverse patient profiles. Such inclusivity not only augments the robustness of research findings but also underscores an ethical imperative to represent and understand the varied experiences of all survivors of stroke.

## Notes

Supplement 1. **eFigure 1.** Flowchart of PRoFESS Cohort Derivation **eFigure 2.** Flowchart of IRIS Cohort Derivation **eFigure 3.** Sankey Diagram Showing the Cross-Sectional Overlap of mRS and NIHSS Categories in the PRoFESS Trial **eFigure 4.** Sankey Diagram Showing the Cross-Sectional Overlap of mRS and NIHSS Categories in the IRIS Trial **eTable 1.** MACE Outcomes in PRoFESS and IRIS by Poststroke Categories of Modified Rankin Scale **eTable 2.** Recurrent Stroke Outcomes in PRoFESS and IRIS by Poststroke Categories of NIH Stroke Scale **eTable 3.** MACE Outcomes in PRoFESS and IRIS by Poststroke Categories of NIH Stroke Scale **eTable 4.** Recurrent Stroke Outcomes in PRoFESS and IRIS by Alternative Poststroke Categories of Modified Rankin Scale **eTable 5.** MACE Outcomes in PRoFESS and IRIS by Alternative Poststroke Categories of Modified Rankin Scale

Supplement 2.
Data Sharing Statement
