# Supplementary material for: Disability and Recurrent Stroke Among Participants in Stroke Prevention Trials
Source: JAMA Netw Open. 2024 Jul 19;7(7):e2423677. doi: 10.1001/jamanetworkopen.2024.23677 (PMC11259901; doi:10.1001/jamanetworkopen.2024.23677)
Supplement: Supplement 1. — eFigure 1. Flowchart of PRoFESS Cohort Derivation eFigure 2. Flowchart of IRIS Cohort Derivation eFigure 3. Sankey Diagram Showing the Cross-Sectional Overlap of mRS and NIHSS Categories in the PRoFESS Trial eFigure 4. Sankey Diagram Showing the Cross-Sectional Overlap of mRS and NIHSS Categories in the IRIS Trial eTable 1. MACE Outcomes in PRoFESS and IRIS by Poststroke Categories of Modified Rankin Scale eTable 2. Recurrent Stroke Outcomes in PRoFESS and IRIS by Poststroke Categories of NIH Stroke Scale eTable 3. MACE Outcomes in PRoFESS and IRIS by Poststroke Categories of NIH Stroke Scale eTable 4. Recurrent Stroke Outcomes in PRoFESS and IRIS by Alternative Poststroke Categories of Modified Rankin Scale eTable 5. MACE Outcomes in PRoFESS and IRIS by Alternative Poststroke Categories of Modified Rankin Scale [file jamanetwopen-e2423677-s001.pdf]

Supplemental Online Content

de Havenon A, Viscoli C, Kleindorfer D, et al. Disability and recurrent stroke among participants in stroke prevention trials. *JAMA Netw Open*. 2024;7(7):e2423677. doi:10.1001/jamanetworkopen.2024.23677

eFigure 1. Flowchart of PRoFESS Cohort Derivation
eFigure 2. Flowchart of IRIS Cohort Derivation
eFigure 3. Sankey Diagram Showing the Cross-Sectional Overlap of mRS and NIHSS Categories in the PRoFESS Trial
eFigure 4. Sankey Diagram Showing the Cross-Sectional Overlap of mRS and NIHSS Categories in the IRIS Trial
eTable 1. MACE Outcomes in PRoFESS and IRIS by Poststroke Categories of Modified Rankin Scale
eTable 2. Recurrent Stroke Outcomes in PRoFESS and IRIS by Poststroke Categories of NIH Stroke Scale
eTable 3. MACE Outcomes in PRoFESS and IRIS by Poststroke Categories of NIH Stroke Scale
eTable 4. Recurrent Stroke Outcomes in PRoFESS and IRIS by Alternative Poststroke Categories of Modified Rankin Scale
eTable 5. MACE Outcomes in PRoFESS and IRIS by Alternative Poststroke Categories of Modified Rankin Scale

This supplemental material has been provided by the authors to give readers additional information about their work.

© 2024 de Havenon A et al. *JAMA Network Open.*

eFigure 1. Flowchart of PRoFESS cohort derivation.

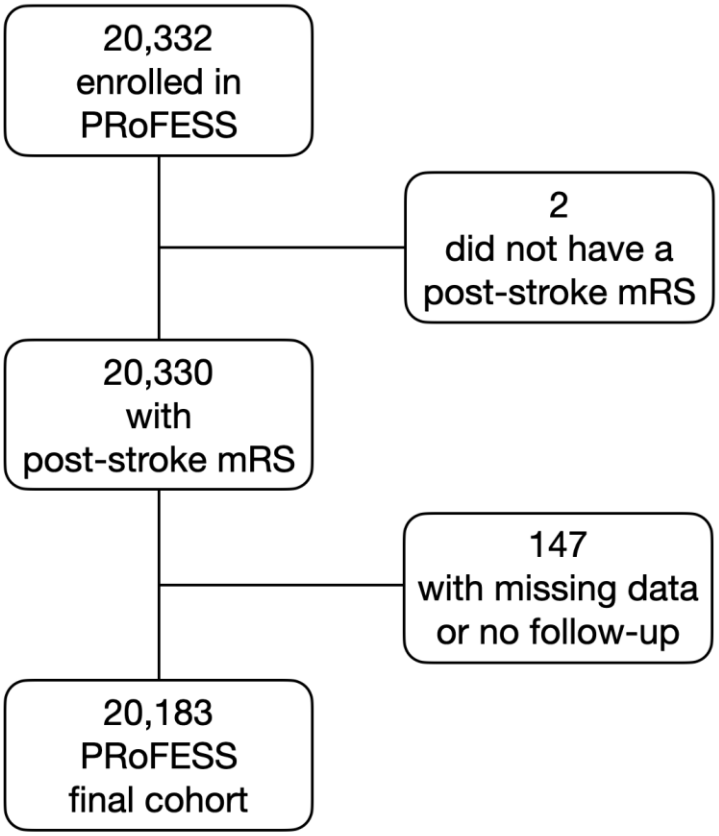

© 2024 de Havenon A et al. *JAMA Network Open.*

eFigure 2. Flowchart of IRIS cohort derivation.

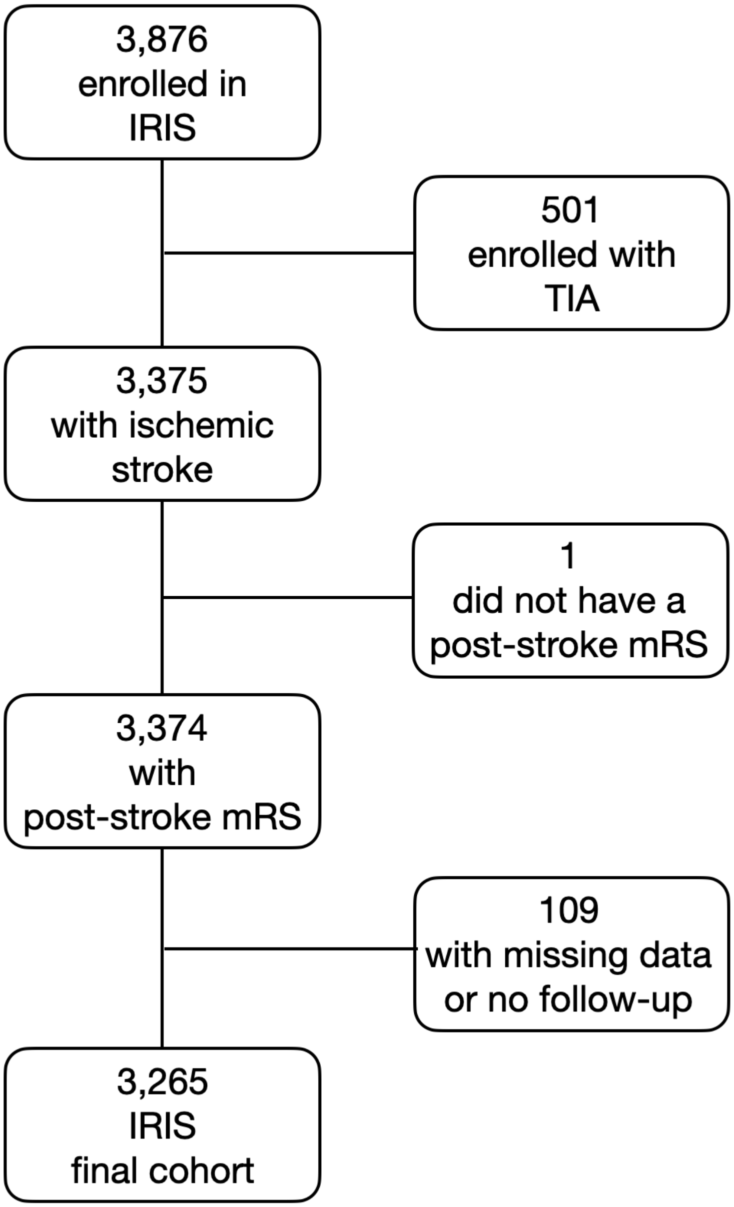

© 2024 de Havenon A et al. *JAMA Network Open.*

eFigure 3. Sankey diagram showing the cross-sectional overlap of mRS and NIHSS categories in the PRoFESS trial.

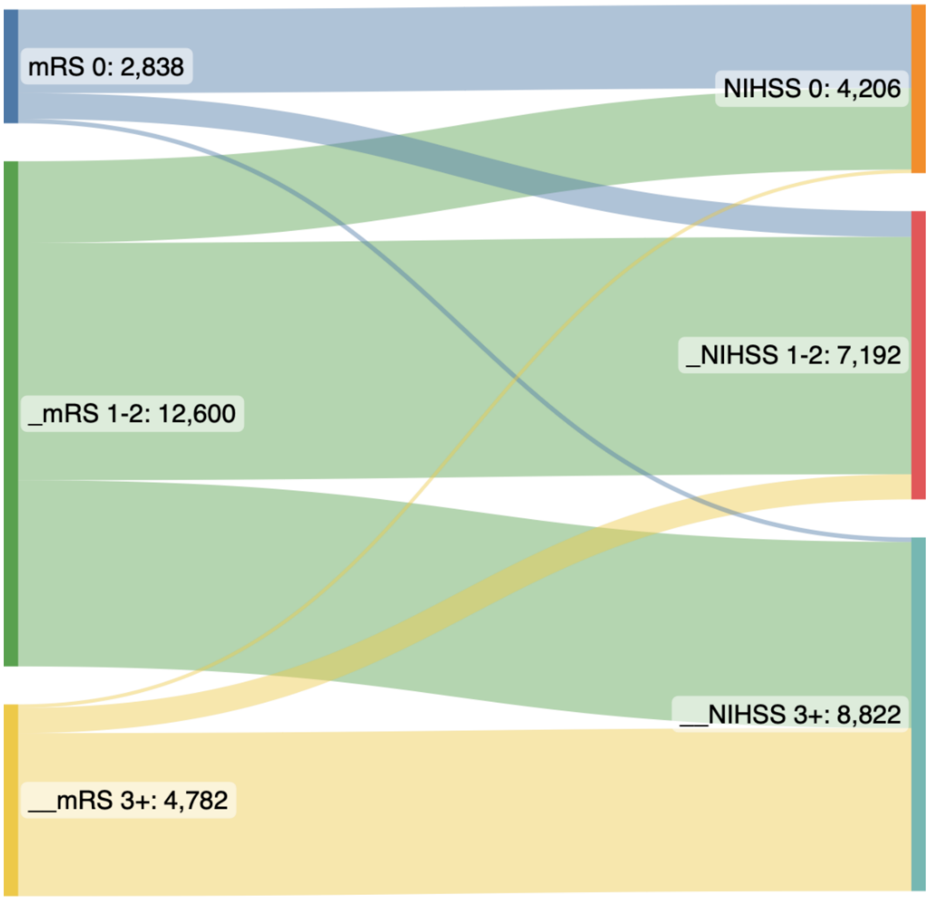

© 2024 de Havenon A et al. *JAMA Network Open.*

eFigure 4. Sankey diagram showing the cross-sectional overlap of mRS and NIHSS categories in the IRIS trial.

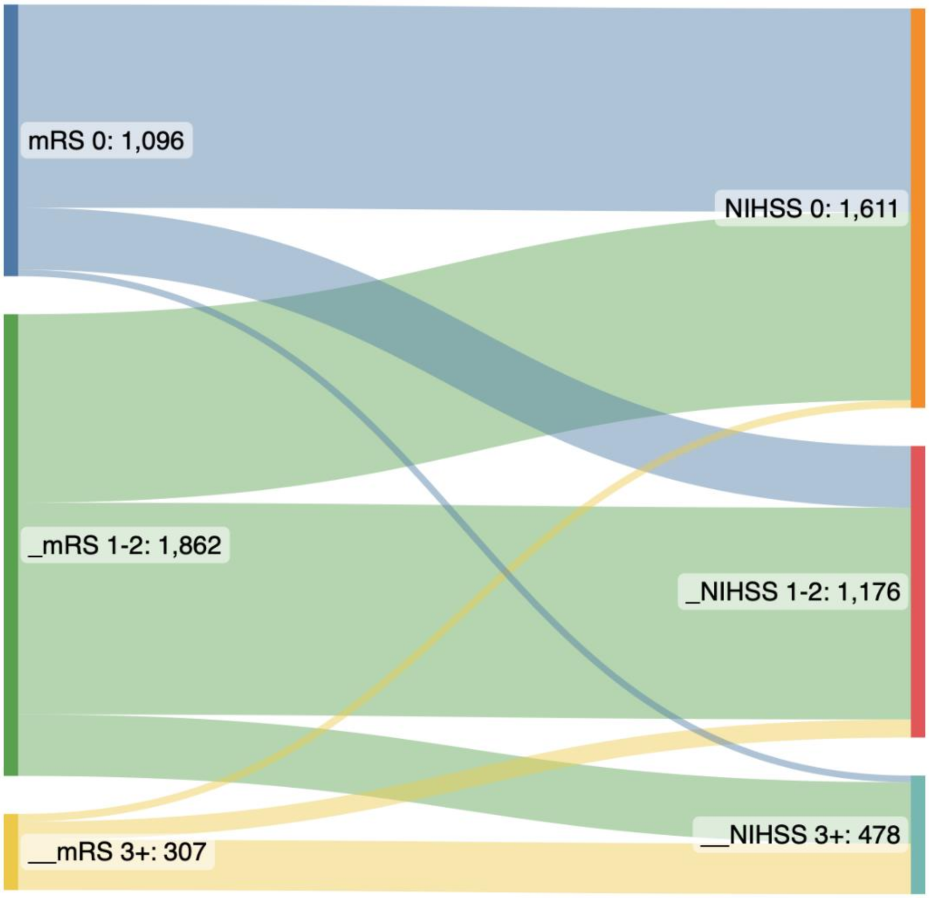

© 2024 de Havenon A et al. *JAMA Network Open.*

eTable 1. MACE outcomes in PRoFESS and IRIS by post-stroke categories of modified Rankin Scale.

	MACE event rate	Unadjusted hazard ratio (95% CI) for MACE*	P value	Adjusted hazard ratio (95% CI) for MACE*	P value
**PRoFESS**					
mRS 0 (n=2,834)	10.1%	Ref	Ref	Ref	Ref
mRS 1-2 (n=12,570)	12.2%	1.27 (1.12-1.44)	<0.001	1.21 (1.06-1.37)	0.004
mRS ≥3 (n=4,779)	17.2%	1.90 (1.66-2.18)	<0.001	1.62 (1.41-1.87)	<0.001
**IRIS****					
mRS 0 (n=1,096)	10.9%	Ref	Ref	Ref	Ref
mRS 1-2 (n=1,862)	13.3%	1.24 (1.00-1.55)	0.052	1.20 (0.98-1.53)	0.113
mRS ≥3 (n=307)	15.3%	1.45 (1.03-2.03)	0.031	1.36 (0.96-1.94)	0.085

*From Cox model adjusted for baseline age, gender, race, country of enrollment, current smoking, atrial fibrillation, diabetes, hypertension, hyperlipidemia, prior stroke, randomization arm, days from qualifying stroke to enrollment, and TOAST classification of the qualifying stroke.

**The proportional hazards assumption was not met for the adjusted model, resulting in a stratified Cox regression with strata for atrial fibrillation, country, and TOAST, after which we met the proportional hazards assumption.

© 2024 de Havenon A et al. *JAMA Network Open.*

eTable 2. Recurrent stroke outcomes in PRoFESS and IRIS by post-stroke categories of NIH Stroke Scale.

	Stroke event rate	Unadjusted hazard ratio (95% CI) for stroke	P value	Adjusted hazard ratio (95% CI) for stroke*	P value
**PRoFESS**					
NIHSS 0 (n=4,206)	7.7%	Ref	Ref	Ref	Ref
NIHSS 1-2 (n=7,192)	8.8%	1.18 (1.04-1.35)	0.014	1.14 (0.99-1.30)	0.062
NIHSS ≥3 (n=8,822)	9.6%	1.33 (1.17-1.51)	<0.001	1.18 (1.04-1.35)	0.013
**IRIS**					
NIHSS 0 (n=1,611)	6.8%	Ref	Ref	Ref	Ref
NIHSS 1-2 (n=1,176)	9.0%	1.44 (1.03-1.76)	0.030	1.27 (0.97-1.67)	0.080
NIHSS ≥3 (n=478)	12.1%	1.84 (1.34-2.53)	<0.001	1.73 (1.24-2.40)	0.001

* Adjusted for baseline age, gender, race, region or country of enrollment, current smoking, atrial fibrillation, hypertension, hyperlipidemia, prior stroke, randomization arm, days from qualifying stroke to enrollment, and TOAST classification of the qualifying stroke. PRoFESS is also adjusted for diabetes.

© 2024 de Havenon A et al. *JAMA Network Open.*

eTable 3. MACE outcomes in PRoFESS and IRIS by post-stroke categories of NIH Stroke Scale.

	MACE event rate	Unadjusted hazard ratio (95% CI) for MACE*	P value	Adjusted hazard ratio (95% CI) for MACE*	P value
**PRoFESS****					
NIHSS 0 (n=4,206)	11.0%	Ref	Ref	Ref	Ref
NIHSS 1-2 (n=7,192)	12.1%	1.13 (1.01-1.26)	0.039	1.10 (0.98-1.23)	0.119
NIHSS ≥3 (n=8,822)	14.9%	1.44 (1.30-1.60)	<0.001	1.32 (1.18-1.47)	<0.001
**IRIS*****					
NIHSS 0 (n=1,611)	11.7%	Ref	Ref	Ref	Ref
NIHSS 1-2 (n=1,176)	12.8%	1.11 (0.90-1.38)	0.340	1.02 (0.83-1.30)	0.873
NIHSS ≥3 (n=478)	15.7%	1.38 (1.06-1.80)	0.019	1.23 (0.93-1.63)	0.148

*From Cox model adjusted for baseline age, gender, race, country of enrollment, current smoking, atrial fibrillation, diabetes, hypertension, hyperlipidemia, prior stroke, randomization arm, days from qualifying stroke to enrollment, and TOAST classification of the qualifying stroke.

**The proportional hazards assumption was not met for the adjusted model, resulting in a stratified Cox regression with strata for atrial fibrillation, treatment arm, and TOAST, after which we met the proportional hazards assumption.

***The proportional hazards assumption was not met for the adjusted model, resulting in a stratified Cox regression with strata for smoking, country, and TOAST, after which we met the proportional hazards assumption.

© 2024 de Havenon A et al. *JAMA Network Open.*

eTable 4. Recurrent stroke outcomes in PRoFESS and IRIS by alternative post-stroke categories of modified Rankin Scale (0-1 vs. 2 vs. ≥3).

	**Stroke event rate**	**Unadjusted hazard ratio (95% CI) for stroke**	**P value**	**Adjusted hazard ratio (95% CI) for stroke***	**P value**
**PRoFESS**					
mRS 0-1 (n=10,357)	8.0%	Ref	Ref	Ref	Ref
mRS 2 (n=5,047)	9.2%	1.19 (1.06-1.33)	0.003	1.11 (0.99-1.25)	0.078
mRS ≥3 (n=4,779)	10.6%	1.42 (1.27-1.59)	<0.001	1.22 (1.09-1.37)	0.001
**IRIS**					
mRS 0-1 (n=2,324)	7.8%	Ref	Ref	Ref	Ref
mRS 2 (n=634)	8.8%	1.12 (0.83-1.51)	0.450	1.08 (0.80-1.46)	0.629
mRS ≥3 (n=307)	11.7%	1.54 (1.08-2.20)	0.018	1.47 (1.02-2.12)	0.038

* Adjusted for baseline age, gender, race, region or country of enrollment, current smoking, atrial fibrillation, hypertension, hyperlipidemia, prior stroke, randomization arm, days from qualifying stroke to enrollment, and TOAST classification of the qualifying stroke. PRoFESS is also adjusted for diabetes.

© 2024 de Havenon A et al. *JAMA Network Open.*

eTable 5. MACE outcomes in PRoFESS and IRIS by alternative post-stroke categories of modified Rankin Scale (0-1 vs. 2 vs. ≥3).

	MACE event rate	Unadjusted hazard ratio (95% CI) for MACE*	P value	Adjusted hazard ratio (95% CI) for MACE*	P value
**PRoFESS**					
mRS 0-1 (n=10,357)	11.2%	Ref	Ref	Ref	Ref
mRS 2 (n=5,047)	13.1%	1.21 (1.10-1.33)	<0.001	1.15 (1.05-1.27)	0.004
mRS ≥3 (n=4,779)	17.2%	1.67 (1.53-1.83)	<0.001	1.46 (1.33-1.60)	<0.001
**IRIS**					
mRS 0-1 (n=2,324)	12.1%	Ref	Ref	Ref	Ref
mRS 2 (n=634)	13.6%	1.13 (0.88-1.43)	0.341	1.09 (0.86-1.40)	0.478
mRS ≥3 (n=307)	15.3%	1.29 (0.95-1.76)	0.103	1.25 (0.91-1.71)	0.171

*From Cox model adjusted for baseline age, gender, race, country of enrollment, current smoking, atrial fibrillation, diabetes, hypertension, hyperlipidemia, prior stroke, randomization arm, days from qualifying stroke to enrollment, and TOAST classification of the qualifying stroke.

© 2024 de Havenon A et al. *JAMA Network Open.*
