# Supplementary material for: Disability and Recurrent Stroke Among Participants in Stroke Prevention Trials
Source: JAMA Netw Open. 2024 Jul 19;7(7):e2423677. doi: 10.1001/jamanetworkopen.2024.23677 (PMC11259901; doi:10.1001/jamanetworkopen.2024.23677)
Supplement: Supplement 2. — Data Sharing Statement [file jamanetwopen-e2423677-s002.pdf]

Data Sharing Statement

de Havenon. Disability and Recurrent Stroke Among Participants in Stroke Prevention Trials. *JAMA Netw Open*. Published July 19, 2024. doi:10.1001/jamanetworkopen.2024.23677

Data
Data available: Yes
Data types: Deidentified participant data
How to access data: Data for the IRIS trial can be requested here: https://www.ninds.nih.gov/current-research/research-funded-ninds/clinical-research/archived-clinical-research-datasets Data for the PRoFESS trial can be requested here: https://search.vivli.org/studyDetails/fromSearch/10ba6524-d58c-4d92-899b-b87a9fc8671a
When available: beginning date: 02-23-2024

Supporting Documents
Document types: None

Additional Information
Who can access the data: Researchers whose proposed use of the data has been approved
Types of analyses: Broadly available to qualified researchers
Mechanisms of data availability: Without investigator support, after approval of a proposal, and with a signed data access agreement
